# CT quantification of pneumonia lesions in early days predicts progression to severe illness in a cohort of COVID-19 patients

**DOI:** 10.7150/thno.45985

**Published:** 2020-04-27

**Authors:** Fengjun Liu, Qi Zhang, Chao Huang, Chunzi Shi, Lin Wang, Nannan Shi, Cong Fang, Fei Shan, Xue Mei, Jing Shi, Fengxiang Song, Zhongcheng Yang, Zezhen Ding, Xiaoming Su, Hongzhou Lu, Tongyu Zhu, Zhiyong Zhang, Lei Shi, Yuxin Shi

**Affiliations:** 1 Department of Radiology, Shanghai Public Health Clinical Center, Fudan University, Shanghai, China; 2 Shanghai Key Laboratory of Artificial Intelligence for Medical Image and Knowledge Graph, Shanghai, China; 3 The SMART (Smart Medicine and AI-based Radiology Technology) Lab, Shanghai Institute for Advanced Communication and Data Science, Shanghai University, Shanghai, China; 4 School of Communication and Information Engineering, Shanghai University, Shanghai, China; 5 Institute of Healthcare Research, Yizhi, Shanghai, China; 6 Shanghai Public Health Clinical Center, Fudan University, Shanghai, China; 7 Department of Severe Hepatology, Shanghai Public Health Clinical Center, Fudan University, Shanghai, China; 8 Department of Infectious Disease, Shanghai Public Health Clinical Center, Fudan University, Shanghai, China; 9 Department of Urology, Shanghai Public Health Clinical Center, Fudan University, Shanghai, China

**Keywords:** COVID-19, Chest CT, Severe illness, Retrospective cohort, Artificial intelligence

## Abstract

**Rationale**: Some patients with coronavirus disease 2019 (COVID-19) rapidly develop respiratory failure or even die, underscoring the need for early identification of patients at elevated risk of severe illness. This study aims to quantify pneumonia lesions by computed tomography (CT) in the early days to predict progression to severe illness in a cohort of COVID-19 patients.

**Methods**: This retrospective cohort study included confirmed COVID-19 patients. Three quantitative CT features of pneumonia lesions were automatically calculated using artificial intelligence algorithms, representing the percentages of ground-glass opacity volume (PGV), semi-consolidation volume (PSV), and consolidation volume (PCV) in both lungs. CT features, acute physiology and chronic health evaluation II (APACHE-II) score, neutrophil-to-lymphocyte ratio (NLR), and d-dimer, on day 0 (hospital admission) and day 4, were collected to predict the occurrence of severe illness within a 28-day follow-up using both logistic regression and Cox proportional hazard models.

**Results**: We included 134 patients, of whom 19 (14.2%) developed any severe illness. CT features on day 0 and day 4, as well as their changes from day 0 to day 4, showed predictive capability. Changes in CT features from day 0 to day 4 performed the best in the prediction (area under the receiver operating characteristic curve = 0.93, 95% confidence interval [CI] 0.87~0.99; C-index=0.88, 95% CI 0.81~0.95). The hazard ratios of PGV and PCV were 1.39 (95% CI 1.05~1.84, *P*=0.023) and 1.67 (95% CI 1.17~2.38, *P*=0.005), respectively. CT features, adjusted for age and gender, on day 4 and in terms of changes from day 0 to day 4 outperformed APACHE-II, NLR, and d-dimer.

**Conclusions**: CT quantification of pneumonia lesions can early and non-invasively predict the progression to severe illness, providing a promising prognostic indicator for clinical management of COVID-19.

## Introduction

In December 2019, coronavirus disease 2019 (COVID-19), caused by severe acute respiratory syndrome coronavirus 2 (SARS-CoV-2), emerged in Wuhan, capital of Hubei province in China 1. The virus quickly spread throughout China and to many other countries/regions, with globally 1,353,361 confirmed cases and 79,235 deaths reported by WHO as of April 8, 2020 2. Most patients of COVID-19 have mild symptoms, but a few could develop severe pneumonia, pulmonary edema, acute respiratory distress syndrome (ARDS), multiple organ dysfunction syndrome or even die. In one epidemical report of COVID-19 by China Center for Disease Control (CDC), among 44,672 confirmed cases, severe patients accounted for 13.8%, and critically ill patients accounted for 4.7% 1. The crude case fatality ratio for critically ill patients was 49.0%, and the average risk of death within a 10-day follow-up was 0.325 for these patients 1. Another recent study revealed that the 28-day case fatality ratio among critically ill patients was as high as 61.5% 3. It is important to unravel the risk factors associated with severe illness and identify patients at an early stage who are most likely to have poor outcomes to focus on prevention and treatment efforts 4.

Several biomarkers have been used to evaluate the severity of patients with infectious pneumonia and to guide clinical interventions, such as the acute physiology and chronic health evaluation II (APACHE-II) score 5, as well as laboratory indicators including neutrophil-to-lymphocyte ratio (NLR) and lactate level 6-8. These clinical biomarkers have been employed to predict prognosis in patients with ARDS 7, 9, 10 or severe acute respiratory syndrome (SARS) 11. However, they are not accurate enough to assess the infection and mostly involve invasive examinations, which may elevate the risk of virus exposure and healthcare-associated infection. Furthermore, the scoring systems like APACHE-II are subjective and time-consuming, which could delay the clinical management against the COVID-19 outbreak. A high level of d-dimer was recently reported as a risk factor for poor outcomes in COVID-19 patients 12, 13. However, the predicting performance of d-dimer has not been studied.

Chest computed tomography (CT) holds great value in screening, diagnosing, and following up COVID-19 patients 14-16. CT assessment has been added as an important criterion for COVID-19 diagnosis and subtyping to the 6^th^ version of national diagnosis and treatment protocols of COVID-19 in China 17. However, currently, CT of COVID-19 is often manually evaluated by radiologists, which is very subjective with large inter- and intra-observer variability thus unable to accurately and quantitatively evaluate the disease severity and is also time-consuming and inefficient. It is now recognized that artificial intelligence (AI) holds promise for deriving quantitative CT features and precisely predicting the risk of lung cancer and poor outcomes of ARDS 10, 18-20. However, to the best of our knowledge, associations between AI-derived CT features quantifying pneumonia lesions and the risk of severe illness in patients with the emerging COVID-19 have not yet been reported. If AI-derived features from CT at an early stage of COVID-19 can be used to predict progression to severe illness, they can be particularly beneficial because CT is noninvasive and easily accessible and AI is time-efficient.

Therefore, this work aimed to investigate the capability of quantitative CT imaging features compared with traditional clinical biomarkers in predicting progression to severe illness in the early stages of COVID-19.

## Materials and Methods

### Patients

This retrospective cohort study was approved by the Ethics Committee of Shanghai Public Health Clinical Center (YJ-2020-S035-01). Informed consent was waived since the study is retrospective and is part of a public health outbreak investigation. As a tertiary hospital for diagnosis and management of infectious diseases and threats against public health for adults and youngsters (i.e., ages ≥14 years) and a WHO designated training organization for new emerging infectious diseases, the Shanghai Public Health Clinical Center is the only designated hospital for treating COVID-19 in Shanghai.

**Figure 1:**
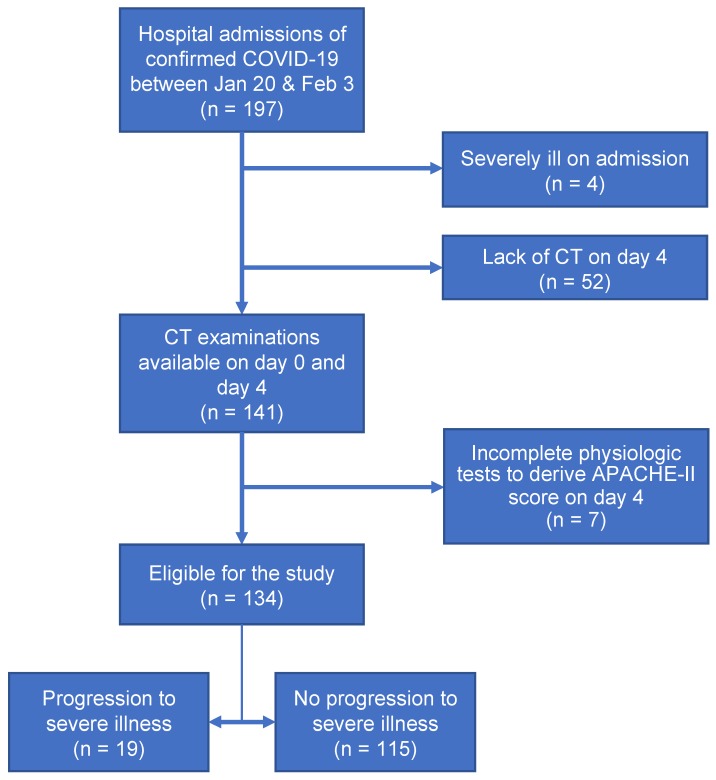
Flow diagram of the study population.

In this study, 197 patients with laboratory-confirmed COVID-19 were admitted to Shanghai Public Health Clinical Center between January 20, 2020 and February 3, 2020. The inclusion criteria of our study were (a) confirmed positive SARS-CoV-2 nucleic acid test by the Shanghai CDC and (b) thin-section CT examinations and laboratory tests on day 0 (the day of admission) and day 4 (4±1 days after admission). Exclusion criteria included (a) severe illness on day 0 or before (n=4), (b) no CT examinations on day 4 (n=52), and (c)incomplete physiologic tests to derive APACHE-II score on day 4 (n=7). Finally, a total of 134 patients with COVID-19 were included in this study. The procedure to enroll patients was conducted, as shown in **Figure 1**.

### Clinical data collection and CT examinations

On day 0 and day 4, records of blood tests, including d-dimer level and NLR, were reviewed for enrolled patients. Also, the APACHE-II score was calculated based on 12 physiologic criteria, age, and previous conditions for each patient.

Chest CT examinations were performed using a 64-slice CT scanner (Hitachi Medical, Japan) without contrast agents on day 0 and day 4. Standard lung algorithm settings were used as follows: 120 kV and automatic tube current (180 mA-400 mA); iterative reconstruction technique; detector, 64 mm; rotation time, 0.35 second; section thickness, 5 mm; collimation, 0.625 mm; pitch, 1.5; matrix, 512×512.

### AI-based quantization of CT images

**Figure 2:**
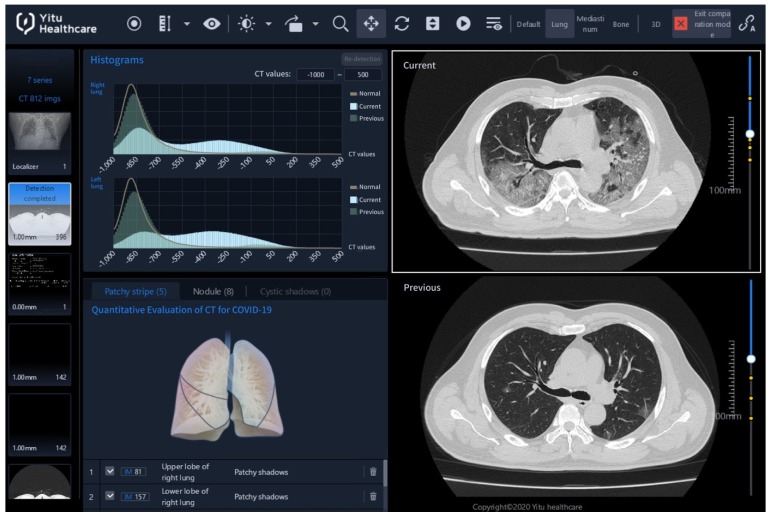
CT image quantization and analysis with artificial intelligence (AI) system. CT images acquired on the day of admission (day 0, in the lower right panel of the figure denoted as “Previous”) and acquired four (±1) days after admission (day 4, upper right denoted as “Current”) can be compared using histograms (upper left) and AI-derived quantitative features. Here, on day 0, the percentage of ground-glass opacity (GGO) volume, percentage of semi-consolidation volume, and percentage of consolidation volume were 0.7, 0.6 and 0.1, while on day 4, they increased to 10.8, 26.1 and 11.5.

As shown in **Figure 2,** the Quantitative Evaluation System of CT for COVID-19 (YT-CT-Lung, YITU Healthcare Technology Co., Ltd., China) was employed as the CT image quantization and analysis tool under supervision of two board-certified radiologists with more than 10 years of experience. The system combined a fully convolutional network with adaptive thresholding and morphological operations for segmentation of lungs and pneumonia lesions 21, 22. External validation with 383 axial CT images from 206 patients showed a Dice coefficient of 82.08% for COVID-19 pneumonia lesion segmentation (unpublished data from our other study with a focus on the development and validation of the AI system). By thresholding on CT values in the pneumonia lesions, three quantitative features were computed, including the percentages of lesion volume with ranges of -700~-500 Hounsfield units (HU), -500~-200 HU, and -200~60 HU. The three AI-derived CT features corresponded to percentages of ground glass opacity (GGO) volume (PGV), semi-consolidation volume (PSV), and consolidation volume (PCV), where semi-consolidation was defined as the area of intermediate homogeneous increase in density 23.

### Endpoint Definition

The endpoint was the severe-event-free survival, which was defined as the time from the date of admission to that of severe illness occurrence (i.e. any severe events). All patients were followed up to the first onset of severe illness, or otherwise for 28 days. According to the guidelines of national diagnosis and treatment protocols for COVID-19 17 and the guidelines of American Thoracic Society 24, severe illness was defined as a condition with any severe event based on one major criterion, two or more minor criteria, or two criteria of additional organ dysfunction, as follows:

(a) Major criteria: respiratory failure requiring mechanical ventilation; shock with the need for vasopressors; extracorporeal membrane oxygenation (ECMO) treatment;

(b) Minor criteria: multilobar infiltrates; respiratory rate ≥ 30 breaths/min; arterial oxygen pressure (PaO_2_) < 60 mmHg; PaO_2_/FiO_2_ ratio ≤ 300 mmHg; oxygen saturation ≤ 93%; hemoptysis 24 h ≥ 100 mL;

(c) Criteria of additional organ dysfunction: multilobar infiltrates; other organ damage. Here, other organ damage covers any one of the following: (c.1) damage to the cardiovascular system: heart function graduation ≥ IV level by New York Heart Association heart function rating; pulmonary arterial hypertension; lower limb thrombosis; severe coma 3~8 points (Glasgow Score); (c.2) acute liver function damage: alanine aminotransferase > 5 times upper limit of normal, according to the liver function test guidelines released by American College of Gastroenterology; (c.3) acute kidney injury: increase in serum creatinine (SCr) to ≥ 2 times baseline, according to Kidney Disease Improving Global Outcomes SCr criteria.

None of the finally included patients had severe illness on day 0. The time when a patient later developed severe illness during the follow up (i.e., the severe-event-free survival) was recorded.

### Statistical Analysis

Patients were divided into two groups according to the severity of illness (severe vs. non-severe). Continuous variables were expressed as the median and interquartile range (IQR) and were compared between groups using the Wilcoxon rank-sum test. Categorical variables were expressed as number and percentage, and Chi-square or Fisher's exact tests were applied for appropriate comparisons between groups. We conducted both binary logistic regression and survival analysis to explore the association between the predictive features and the development of severe illness.

Multivariate logistic regression models were constructed to make binary predictions for the adverse outcomes (i.e. severe or non-severe) using APACHE-II, NLR, d-dimer, CT features, and NLR combined with all CT features (denoted as NLR+CT features). The prediction performance was estimated and reported with the area under the receiver operating characteristic (ROC) curve (AUC). All logistic models were adjusted for traditional clinical variables including age and gender. ROC comparisons were performed using DeLong's method.

For survival analysis, Kaplan-Meier survival curves and log-rank analyses were first used to analyze the individual effects of CT features, APACHE-II, NLR, and d-dimer on the severe-event-free survival. In Kaplan-Meier survival analysis, each variable was binarized by the median. Subsequently, multivariate Cox proportional hazard models were built for APACHE-II, NLR, d-dimer, CT features, and NLR+CT features, with age and gender considered as potential confounders. The performance of the Cox proportional hazard model was evaluated with the concordance index (C-index).

All analyses were conducted with R software version 3.6.2 (R Foundation for Statistical Computing, Vienna, Austria). A two-tailed *P*-value less than 0.05 was considered as statistically significant.

## Results

### Basic characteristics of patients

Demographic and clinical characteristics of 134 patients with COVID-19 are enumerated in **Table 1**. The median age was 51.5 years (IQR 37.0~65.0; range 15.0~80.0), and 63 (47.0%) patients were male. For epidemic exposure history to the source of transmission, recent travel to Hubei, contact with people from Hubei, and close contact with confirmed patients were documented in 64.2%, 11.2%, and 12.7% of patients, respectively. Fever (81.3%) and cough (39.6%) were the most common symptoms, and there were 4 (3.0%) asymptomatic patients.

No patients were lost to follow-up. A total of 19 (14.2%) patients progressed to severe illness during the follow-up, among whom 6 (31.6%) met the major criteria of severe illness, 10 (52.6%) met the minor criteria, and 3 (15.8%) met the criteria of additional organ dysfunction. The median time from admission to the occurrence of severe illness was 9 days (IQR 6.5~12.5; range 3.0~16.0). The median duration from admission to the occurrence of severe illness that met the major criteria, the minor criteria and the additional organ dysfunction were 9.5 days (range 5.0~14.0), 7.0 days (range 3.0~16.0) and 10.0 days (range 4.0~11.0), respectively.

Severe (63.0 years, IQR 40.0~65.5) patients were older than the non-severe (50.0 years, IQR 36.0~64.0), but the difference was not of statistical significance (*P*=0.086). There were significantly more males in the severe group than the non-severe group (78.9% vs 41.7%, *P*=0.006). The highest temperature was significantly higher in the severe group (38.5 ℃, IQR 38.0~38.8) than the non-severe group (38.0 ℃, IQR 37.4~38.4, *P*=0.015). Other demographic and clinical variables listed in **Table 1** showed no significant differences between the two groups (all *P*>0.05). The median time from symptom onset to admission was 4.0 days (IQR: 2.0~7.0; range:0~20.0) in the severe group, while that was 6.0 days (IQR: 3.5~7.5; range:1.0~14.0) in the non-severe group. Days from symptom onset to admission were not significantly different between the two groups (*P*=0.176).

### CT features, APACHE-II, NLR, and d-dimer in the severe and non-severe groups

Comparisons of CT features, APACHE-II, NLR and d-dimer between severe and non-severe patients on day 0, day 4 and their changes from day 0 to day 4 are depicted in **Supplemental Table S1**.

On day 0, significant differences were observed for all three CT features between severe and non-severe patients (all *P*<0.01). NLR was significantly higher in the severe group than in the non-severe group (*P*=0.010). D-dimer levels were also higher in the sever group (*P*=0.011). However, APACHE-II exhibited no significant differences between the two groups (*P*=0.518).

On day 4, all three CT features were significantly higher in severe patients than in non-severe patients (all *P*<0.001). APACHE-II (*P*=0.019), NLR (*P*<0.001) and d-dimer (*P*=0.003) were also significantly higher in the severe group than in the non-severe group.

Regarding the changes from day 0 to day 4, all CT features showed a more distinct increase in the severe patients (all *P*<0.001), while the clinical biomarkers (e.g. APACHE-II, NLR, and d-dimer) showed no significant increase.

**Figure 3:**
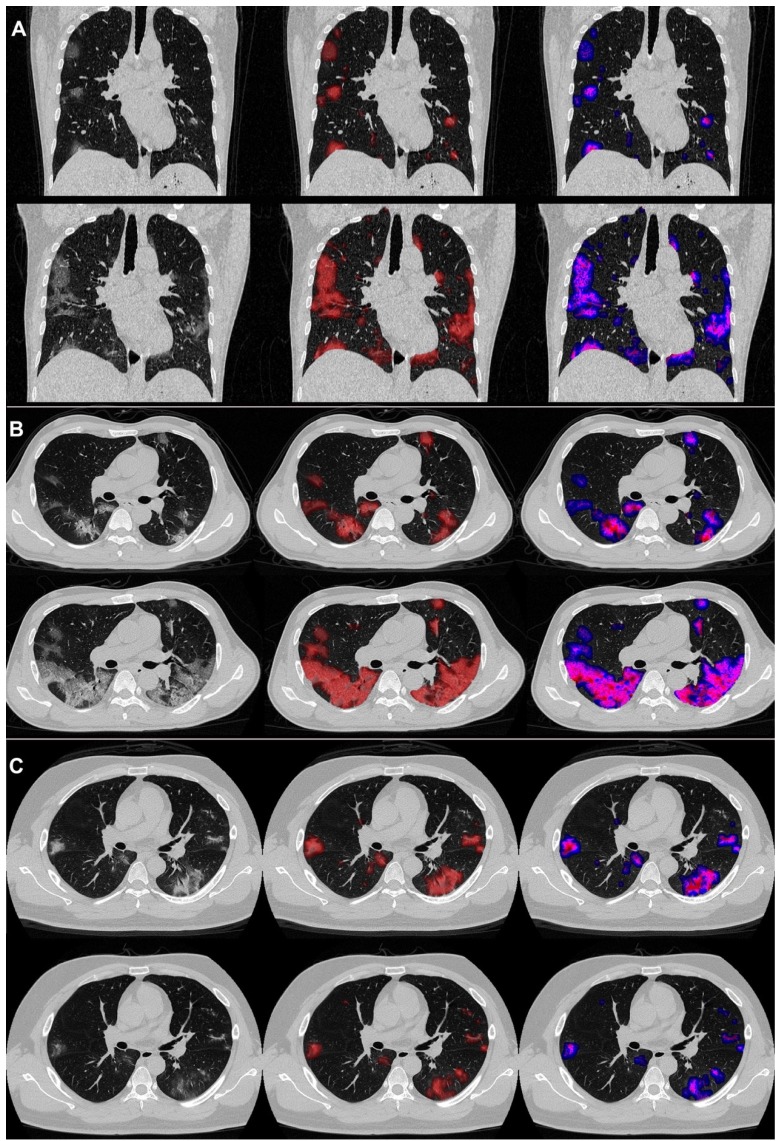
COVID-19 pneumonia lesions detected by the AI system and visualized as pseudo colors. First to third columns: initial CT images; displayed with red pseudo colors; displayed with blue, pink, and red pseudo colors representing ground-glass opacity (GGO), semi-consolidation and consolidation, respectively. Pictures of two patients are illustrated: one was a 38year-old male (A and B), who reached the endpoint of progression to severe illness after 7 days from admission, and the other was a 31-year-old male (C), who did not meet the endpoint during the follow-up and was discharged from the hospital after 13 days from admission. The upper halves of A, B, and C show images on day 0, and the lower halves show images on day 4.

An example of the differences in CT manifestations between the severe and non-severe groups is presented in **Figure 3** displaying CT images of two male patients, who were under 40 years old. One patient developed severe illness, while the other did not, and got discharged meeting the following discharge criteria: two consecutive negative COVID-19 nucleic acid detections at least 24 h apart, afebrile for more than 3 days, and respiratory symptoms significantly relieved. The average PGV, PSV and PCV in the non-severe patients decreased from 1.4, 1.3 and 0.5 on day 0 to 1.0, 0.5 and 0.1 on day 4. On the contrary, the average PGV, PSV, and PCV of the patients in the severe group increased from 3.0, 2.7 and 1.1 on day 0 to 8.3, 11.8 and 5.4 on day 4. Detailed changes in the volumes and the percentages were captured by the AI-derived CT features.

### Relationships between CT features and severe illness

The prediction performance of COVID-19 severe illness using CT features, APACHE II, NLR, d-dimer, and NLR+CT features were determined based on logistic regression and ROC analysis. AUCs are shown in **Table 2**, and ROC curves are also presented in **Supplemental Figure S1**.

On day 0, all models (i.e. APACHE-II, NLR, d-dimer, and CT features) achieved AUCs slightly below 0.80, showing moderate ability in discriminating the severe from the non-severe group, and the performances were close to each other (all *P*>0.05 when compared to the model using CT features). By day 4, all models except for APACHE-II showed improved performance, with the AUCs of NLR, d-dimer, and CT features increasing to 0.84 (95% CI 0.75~0.93), 0.78 (95% CI 0.67~0.88) and 0.89 (95% CI 0.80~0.97), respectively. As for the changes from day 0 to day 4, CT features demonstrated elevated discriminative capability (AUC=0.93, 95% CI 0.87~0.99), which was significantly better than that for APACHE-II (AUC=0.82, 95% CI 0.72~0.91, *P*=0.046), NLR (AUC=0.78, 95% CI 0.67~0.88, *P*=0.001), and d-dimer (AUC=0.78, 95% CI 0.67~0.88, *P*=0.001). As for day 4 and the changes from day 0 to day 4, the models with PSV or PCV were comparable to that of all three CT features (both *P*>0.05) while the model with PGV was inferior (both *P*<0.05). When NLR was added to CT features, there was no significant improvement for all three data points (all *P*>0.1).

### Relationship between CT features and severe-event-free survival

As displayed in **Supplemental Figure S2-4**, the Kaplan-Meier analyses showed that for all CT features and APACHE-II, the values on day 4 and the changes from day 0 to day 4 were significantly and negatively associated with severe-event-free survival. In contrast, NLR and d-dimer on day 0 and day 4 showed a significant and inverse association with severe-event-free survival.

The results of the multivariate Cox models are presented in** Table 3**. On day 0, all models showed a moderate performance to predict severe-event-free survival, with all C-indices slightly lower than 0.80. Compared to day 0, by day 4, the C-index of the CT features increased to 0.85 (95% CI 0.76~0.95), which was significantly (both *P*<0.001) better than those of APACHE-II (0.76, 95% CI 0.64~0.87), NLR (0.77, 95% CI 0.67~0.86) and d-dimer (0.76, 95% CI 0.66~0.86). The model of changes from day 0 to day 4 yielded C-index of 0.88 (95% CI 0.81~0.95) in CT features, and significantly outperformed those (all *P*<0.001) for APACHE-II (0.80, 95% CI 0.70~0.90), NLR (0.76, 95% CI 0.66~0.85) and d-dimer (0.76, 95% CI 0.66~0.86). On day 4, the models with PSV or PCV were comparable to that of all three CT features (both *P*>0.05), while the model with PGV was inferior (both *P*<0.05). As for the changes from day 0 to day 4, all models with only one of the three CT features performed worse than the model with all CT features (all *P*<0.05). Adding NLR to the CT features showed no improvement for all three data points (all *P*>0.1).

Hazard ratio (HR) estimates for variables included in each multivariate Cox regression model are displayed in **Supplemental Figure S5-7**. Specifically, after considering the age and gender, significant inverse associations with severe-event-free survival remained for the changes in PGV (HR=1.39, 95% CI 1.05~1.84, *P*=0.023) from day 0 to day 4 as well as those in PCV (HR=1.67, 95% CI 1.17~2.38, *P*=0.005).

## Discussion

To our knowledge, this is the first cohort study to predict outcomes in patients with COVID-19 using noninvasive quantitative CT measurements. Three CT features representing the lesion volume ratios of GGO, semi-consolidation, and consolidation were automatically quantified with AI. Our results showed that CT features on day 0 and day 4, as well as their changes from day 0 to day 4, could predict risk of COVID-19 patients progressing to severe illness. In particular, the changes in CT features from day 0 to day 4 performed best in the prediction. Furthermore, CT features outperformed the traditional clinical biomarkers including APACHE-II, NLR, and d-dimer levels on day 4 and with the changes from day 0 to day 4 regardless of adjustment of age and gender.

We chose to investigate the potential features in predicting severe illness from three data points on day 0 (the date of hospital admission), day 4 (4 days later after admission), and the changes from day 0 to day 4. Such research design was not applied previously where only static measures of one time point were explored 12. In this study, we found that CT features on day 4 performed much better than that on day 0, which is consistent with our previous report that some COVID patients present dramatic changes on CT imaging on day 4 compared to day 0 15. Interestingly, the changes in CT features from day 0 to day 4 showed the best performance in predicting severe illness. This observation suggested that the dynamic trends in CT manifestation changes are extremely valuable in predicting poor outcomes of COVID-19, an implication that might also apply to other diseases.

Given the previously reported prognostic potential of NLR and d-dimer 6, 25 and the feasibility for routine blood analysis, we adopted them as representative laboratory biomarkers for comparison in this study. Though the lactate level might also be a useful laboratory indicator, it was not widely available and thus was not investigated here. Our findings showed that the NLR and d-dimer were inferior to CT features in predicting the severity of COVID-19 and the combination of these markers with CT features did not significantly outperform the model with CT features alone.

There are a few limitations of this study that identify areas for future work. First, the study did not consider the treatment as a factor for prognostic prediction. However, no specialized therapeutics have been identified for COVID-19 so far, and currently the mainstay for its treatments are limited to supportive care. For the patients at our Center, several treatments were adopted including oxygen therapy, mechanical ventilation, ECMO, antiviral treatment, antibiotic treatment, glucocorticoids, and intravenous immunoglobulin therapy. Since all patients were treated in the same hospital, it is reasonable to assume that treatment variations might be negligible between the severe and non-severe groups. In the future, the comparison of outcomes of various treatments is needed for response prediction.

Second, radiomics, an AI technique that automatically extracts a large number of quantitative features from medical images for diagnosis or prognosis, has emerged in cancer research 26, 27. It may also be applicable to CT images of COVID-19 and holds future promise.

Third, the endpoint in this study was progression to severe illness, and until the follow-up deadline, there were no deaths among the enrolled patients. In a recently published work by Zhou et al, older age, higher SOFA score, and d-dimer greater than 1μg/mL were found to be associated with an elevated risk of death 12. However, this study did not explore the potential value of imaging in predicting the risk of poor outcomes in COVID-19. Therefore, future studies would further enhance risk stratification by incorporating dynamic monitoring of traditional clinical as well as radiological measurements and using an endpoint of death.

Finally, all CT images studied in this work were acquired on the same CT scanner (Hitachi Medical, Japan) in one clinical center. Extensive research with data from multi-sites and various scanners is warranted to validate the findings of this study.

## Conclusions

In this cohort study, by using AI algorithms, we have shown that three quantitative volume ratios of COVID-19 lung lesions on CT scans are superior to previous clinical biomarkers including APACHE-II, NLR, and d-dimer levels, and are a novel and promising predictor of COVID-19 progression to severe illness. These CT features may provide clinicians with useful early prognostic information to facilitate pretreatment risk stratification for COVID-19, and guide the medical staff to conduct more intensive surveillance and treatment to patients at high risk of severe illness to improve outcomes. Future large-scale prospective studies are warranted to validate these CT features in predicting severe illness development and other important outcomes in COVID-19.

## Supplementary Material

Supplementary figures and table.

## Figures and Tables

**Table 1:** Demographic and clinical characteristics of the patients

	All patients (n=134)	Severe (n=19)	Non-severe (n=115)	*P*
**Age, year (IQR)**	51.5(37.0~65.0)	63.0(40.0~65.5)	50.0(36.0~64.0)	0.086
**Male, n (%)**	63(47.0%)	15(78.9%)	48(41.7%)	0.006
**Exposure to the source of transmission, n (%)**				0.297
Never been to Hubei	12(9.0%)	3(15.8%)	9(7.8%)	
Recently been to Hubei	86(64.2%)	10(52.6%)	76(66.1%)	
Contacted with people from Hubei	15(11.2%)	1(5.3%)	14(12.2%)	
Contacted with patients	17(12.7%)	4(21.1%)	13(11.3%)	
**Symptoms, n (%)**				
No symptoms	4(3.0%)	0(0.0%)	4(3.5%)	1.000
Fever	109(81.3%)	18(94.7%)	91(79.1%)	0.199
Highest temperature, ℃ (IQR)	38.0(37.5~38.5)	38.5(38.0~38.8)	38.0(37.4~38.4)	0.015
Cough	53(39.6%)	10(52.6%)	43(37.4%)	0.315
Sputum production	24(17.9%)	5(26.3%)	19(16.5%)	0.334
Shortness of breath	3(2.2%)	0(0.0%)	3(2.6%)	1.000
Headache and dizziness	8(6.0%)	1(5.3%)	7(6.1%)	1.000
Sore throat	7(5.2%)	0(0.0%)	7(6.1%)	0.593
Fatigue	26(19.4%)	3(15.8%)	23(20.0%)	1.000
Poor appetite	13(9.7%)	2(10.5%)	11(9.6%)	1.000
Sore muscle	13(9.7%)	0(0.0%)	13(11.3%)	0.213
Diarrhea	6(4.5%)	0(0.0%)	6(5.2%)	0.594
Chest distress	7(5.2%)	1(5.3%)	6(5.2%)	1.000
Chill	5(3.7%)	0(0.0%)	5(4.3%)	1.000
**Pre-existing conditions**				
Hypertension	27(20.1%)	6(31.6%)	21(18.3%)	0.217
Diabetes	10(7.5%)	3(15.8%)	7(6.1%)	0.152
Coronary heart disease	5(3.7%)	1(5.3%)	4(3.5%)	0.540
**Days from symptom onset to admission**	4.0(2.0~7.0)	6.0(3.5~7.5)	4.0(2.0~7.0)	0.176

Note: IQR: Interquartile Range.

**Table 2:** Performance for predicting progression to severe illness with logistic regression analysis

	Day 0	*P*	Day 4	*P*	Changes from Day 0 to Day 4	*P*
**APACHE-II**	0.78(0.69~0.88)	0.554	0.77(0.66~0.89)	0.076	0.82(0.72~0.91)	0.046
**NLR**	0.78(0.67~0.88)	0.636	0.84(0.75~0.93)	0.156	0.78(0.67~0.88)	0.001
**D-dimer**	0.75(0.64~0.85)	0.410	0.78(0.67~0.88)	0.007	0.78(0.67~0.88)	0.001
**PGV**	0.76(0.65~0.86)	0.753	0.83(0.73~0.93)	0.015	0.84(0.74~0.93)	0.015
**PSV**	0.76(0.65~0.86)	0.644	0.87(0.77~0.97)	0.256	0.92(0.86~0.99)	0.464
**PCV**	0.76(0.66~0.86)	0.738	0.88(0.79~0.97)	0.572	0.91(0.85~0.98)	0.190
**CT features**	0.76(0.66~0.86)	Reference	0.89(0.80~0.97)	Reference	0.93(0.87~0.99)	Reference
**NLR + CT features**	0.78(0.67~0.88)	0.551	0.89(0.80~0.97)	0.432	0.93(0.87~0.99)	0.336

Note: (a) Results are presented as the area under the receiver operating characteristic curve (AUC) along with 95% CI. (b) PGV=Percentage of GGO volume; PSV=Percentage of semi-consolidation volume; PCV=Percentage of consolidation volume. (c) All models were adjusted for traditional clinical variables including age and gender.

**Table 3:** Performance for predicting severe-event-free survival with Cox proportional hazard models

	Day 0	*P*	Day 4	*P*	Changes from Day 0 to Day 4	*P*
**APACHE-II**	0.77(0.68~0.85)	0.768	0.76(0.64~0.87)	<0.001	0.80(0.70~0.90)	<0.001
**NLR**	0.75(0.66~0.84)	0.930	0.77(0.67~0.86)	<0.001	0.76(0.66~0.85)	<0.001
**D-dimer**	0.73(0.64~0.83)	0.422	0.76(0.66~0.86)	<0.001	0.76(0.66~0.86)	<0.001
**PGV**	0.74(0.64~0.83)	0.610	0.80(0.70~0.89)	<0.001	0.81(0.72~0.90)	<0.001
**PSV**	0.74(0.64~0.83)	0.870	0.84(0.74~0.94)	0.216	0.88(0.81~0.95)	0.022
**PCV**	0.75(0.66~0.84)	0.965	0.86(0.77~0.95)	0.477	0.88(0.80~0.95)	0.049
**CT features**	0.75(0.66~0.84)	Reference	0.85(0.76~0.95)	Reference	0.88(0.81~0.95)	Reference
**NLR + CT features**	0.75(0.66~0.84)	0.312	0.86(0.77~0.95)	0.543	0.88(0.81~0.95)	0.717

Note: (a) Results are presented as concordance indices (95% CI). (b) PGV=Percentage of GGO volume; PSV=Percentage of semi-consolidation volume; PCV=Percentage of consolidation volume. (c) All models were adjusted for traditional clinical variables including age and gender.

## Abbreviations

AI: artificial intelligence
APACHE-II: acute physiology and chronic health evaluation II
ARDS: acute respiratory distress syndrome
AUC: area under the ROC curve
CI: confidence interval
C-index: concordance index
COVID-19: coronavirus disease 2019
CT: computed tomography
ECMO: extracorporeal membrane oxygenation
GGO: ground-glass opacity
HR: hazard ratio
HU: Hounsfield units
IQR: interquartile range
NLR: neutrophil-to-lymphocyte ratio
PaO_2_: arterial oxygen pressure
PCV: percentage of consolidation volume
PGV: percentage of GGO volume
PSV: percentage of semi consolidation volume
ROC: receiver operating characteristic
SARS: severe acute respiratory syndrome
SARS-CoV-2: severe acute respiratory syndrome coronavirus 2
SCr: serum creatinine
