# Supplementary material for: CT quantification of pneumonia lesions in early days predicts progression to severe illness in a cohort of COVID-19 patients
Source: Theranostics. 2020 Apr 27;10(12):5613–22. doi: 10.7150/thno.45985 (PMC7196293; doi:10.7150/thno.45985)
Supplement: Supplementary file 1 — Supplementary figures and table. [file thnov10p5613s1.pdf]

Supplemental Figures and Tables

Table S1. Comparisons of clinical biomarkers and quantitative CT features for the patients who progressed to severe illness (severe group) versus those who did not progress to severe illness (non-severe group).

	**Day 0**			**Day 4**			**Changes from Day 0 to Day 4**		
	**Severe (n=19)**	**Non-severe (n=115)**	***P***	**Severe (n=18)**	**Non-severe (n=115)**	***P***	**Severe (n=18)**	**Non-severe (n=115)**	***P***
**Clinical biomarkers**									
APACHE-II	9.0(4.0~11.0)	8.0(5.0~11.0)	0.518	11.5(7.5~13.8)	8.0(4.0~11.0)	0.019	5.0(0.2~8.5)	0.0(-1.5~2.0)	0.003
NLR	3.4(2.8~5.8)	2.7(1.8~3.7)	0.010	5.0(2.4~9.7)	1.9(1.5~2.6)	<0.001	1.2(-0.9~3.0)	-0.4(-1.5~0.2)	0.005
D-dimer	0.5(0.3~0.9)	0.3(0.2~0.5)	0.011	0.8(0.4~1.0)	0.4(0.3~0.6)	0.003	0.2(0.0~0.3)	0.0(-0.0~0.1)	0.045
**CT features**									
PGV, %	2.2(0.9~4.2)	0.8(0.2~2.1)	0.009	5.5(4.8~8.8)	1.5(0.7~2.8)	<0.001	3.7(1.4~5.0)	0.4(0.0~1.3)	<0.001
PSV, %	2.4(0.7~4.6)	0.7(0.1~2.1)	0.005	11.4(5.9~16.3)	1.2(0.6~2.9)	<0.001	5.5(3.8~10.9)	0.3(0.0~1.2)	<0.001
PCV, %	1.1(0.2~2.2)	0.3(0.0~1.0)	0.009	5.8(2.3~11.3)	0.7(0.2~1.5)	<0.001	4.0(1.8~7.5)	0.2(0.0~0.7)	<0.001

Note:

(a) All variables are expressed as median (interquartile range). GGO, ground glass opacity.

(b) PGV=Percentage of GGO volume; PSV=Percentage of semiconsolidation volume; PCV=Percentage of consolidation volume

(c) One patient had severe events on the same day when performing the second CT, and thus he was removed from the analysis with day 4 and changes from day 0 to day 4.

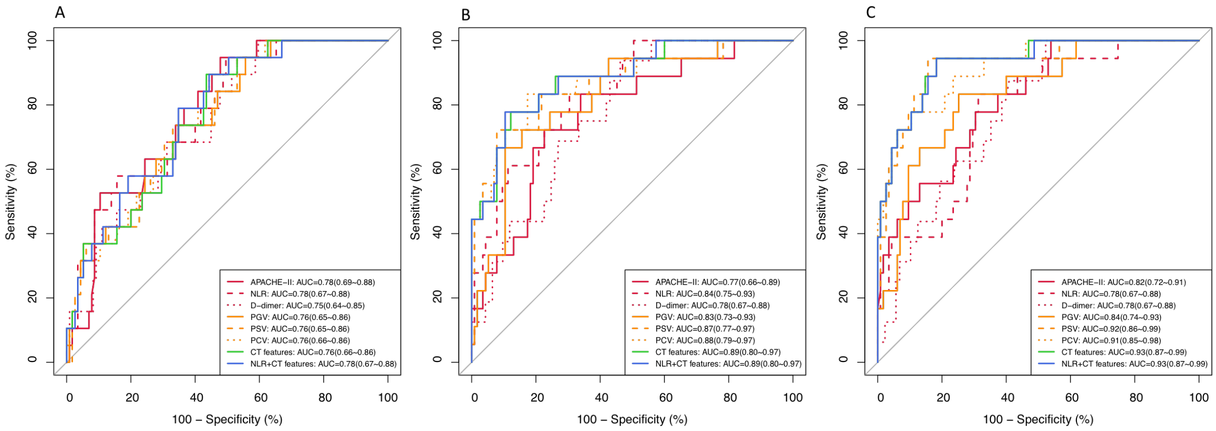

Figure S1. Receiver operating characteristic (ROC) curves for predicting occurrence of severe illness with logistic regression analysis. A: on day 0. B: on day 4. C: in terms of changes from day 0 to day 4.

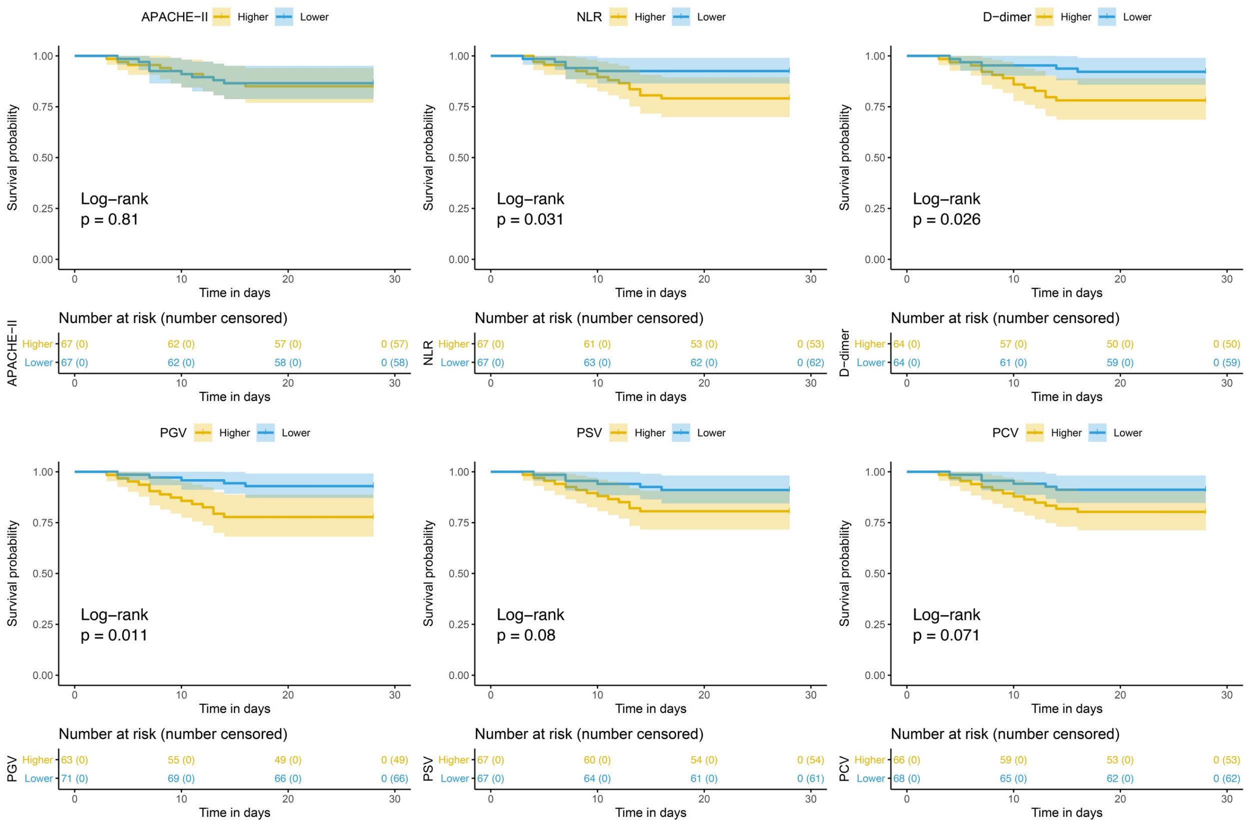

Figure S2 Kaplan-Meier survival analysis of the relationships between each of NLR, APACHE II, and quantitative CT features and severe-event-free survival on day 0. Each feature was stratified by the median to perform Kaplan-Meier analysis.

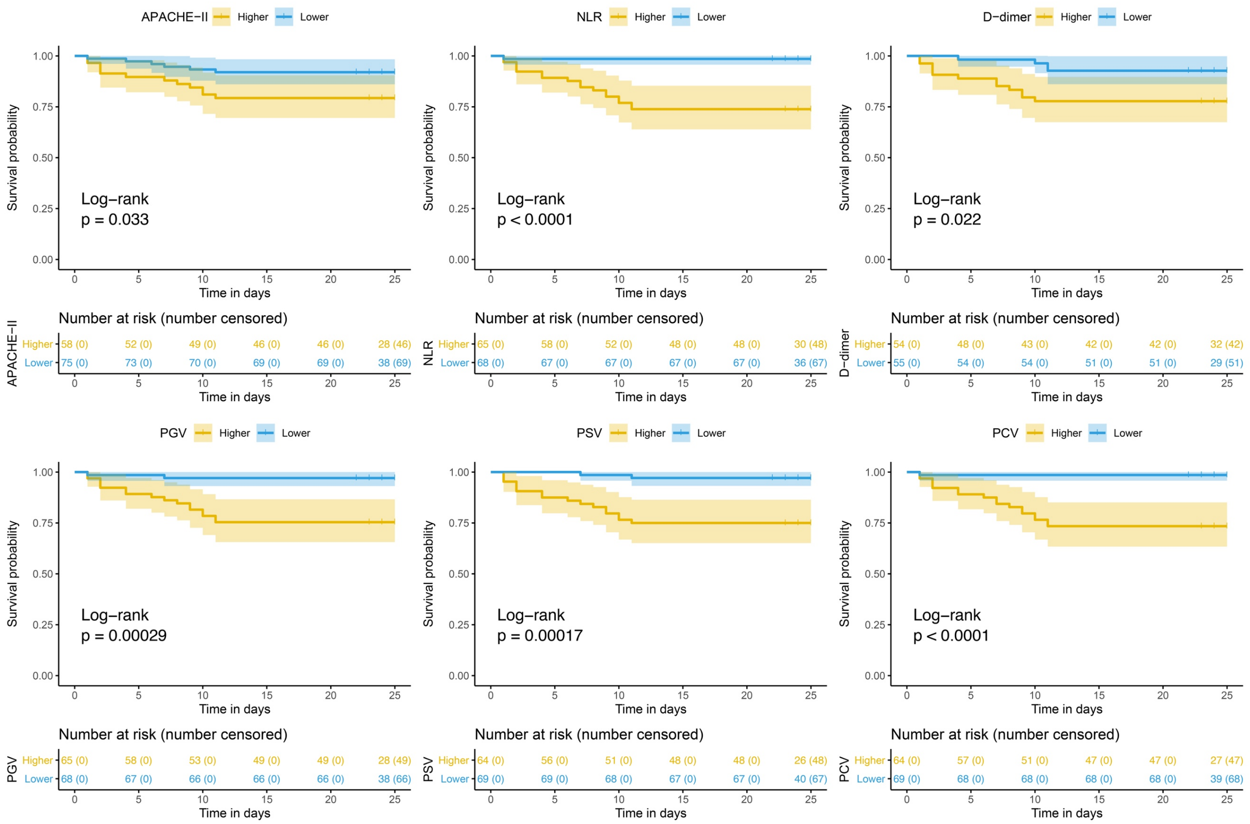

Figure S3. Kaplan-Meier survival analysis of the relationships between each of NLR, APACHE II, and quantitative CT features and severe-event-free survival on day 4. Each feature was stratified by the median to perform Kaplan-Meier analysis.

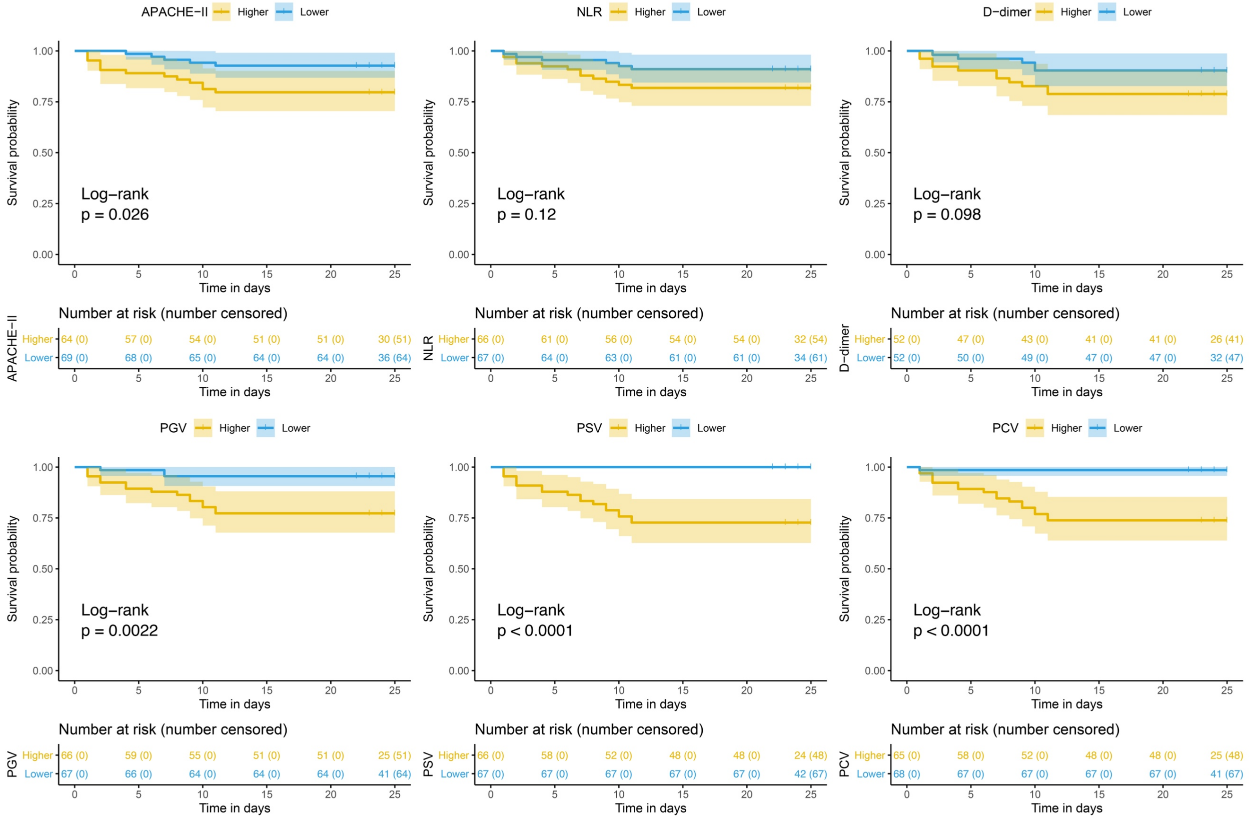

Figure S4. Kaplan-Meier survival analysis of the relationships between each of NLR, APACHE II, and quantitative CT features and severe-event-free survival in terms of changes from day 0 to day 4. Each feature was stratified by the median to perform Kaplan-Meier analysis.

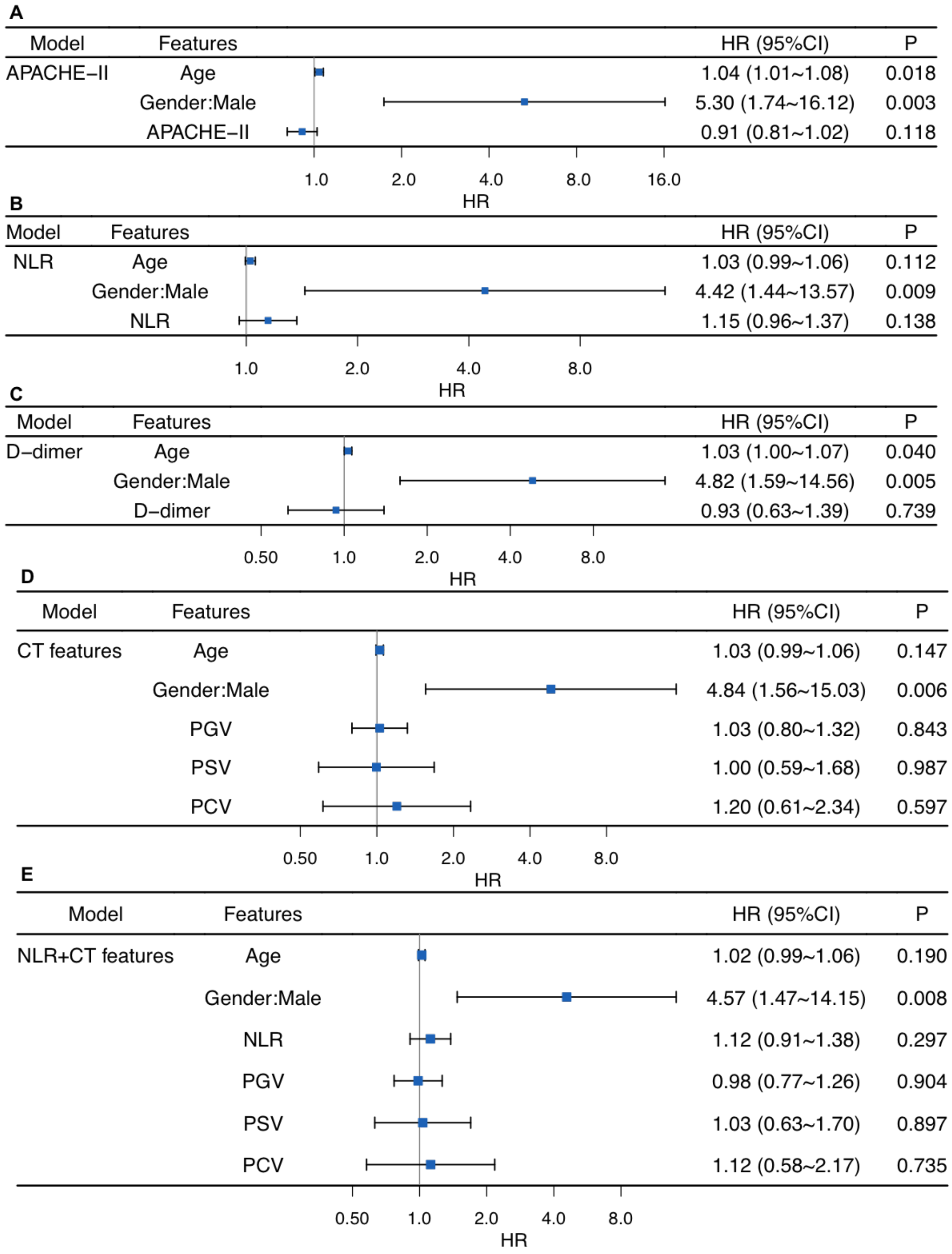

Figure S5. Relationship between each feature on day 0 and severe-event-free survival on the multivariate Cox analyses. All models were adjusted for traditional clinical variables including age and gender.

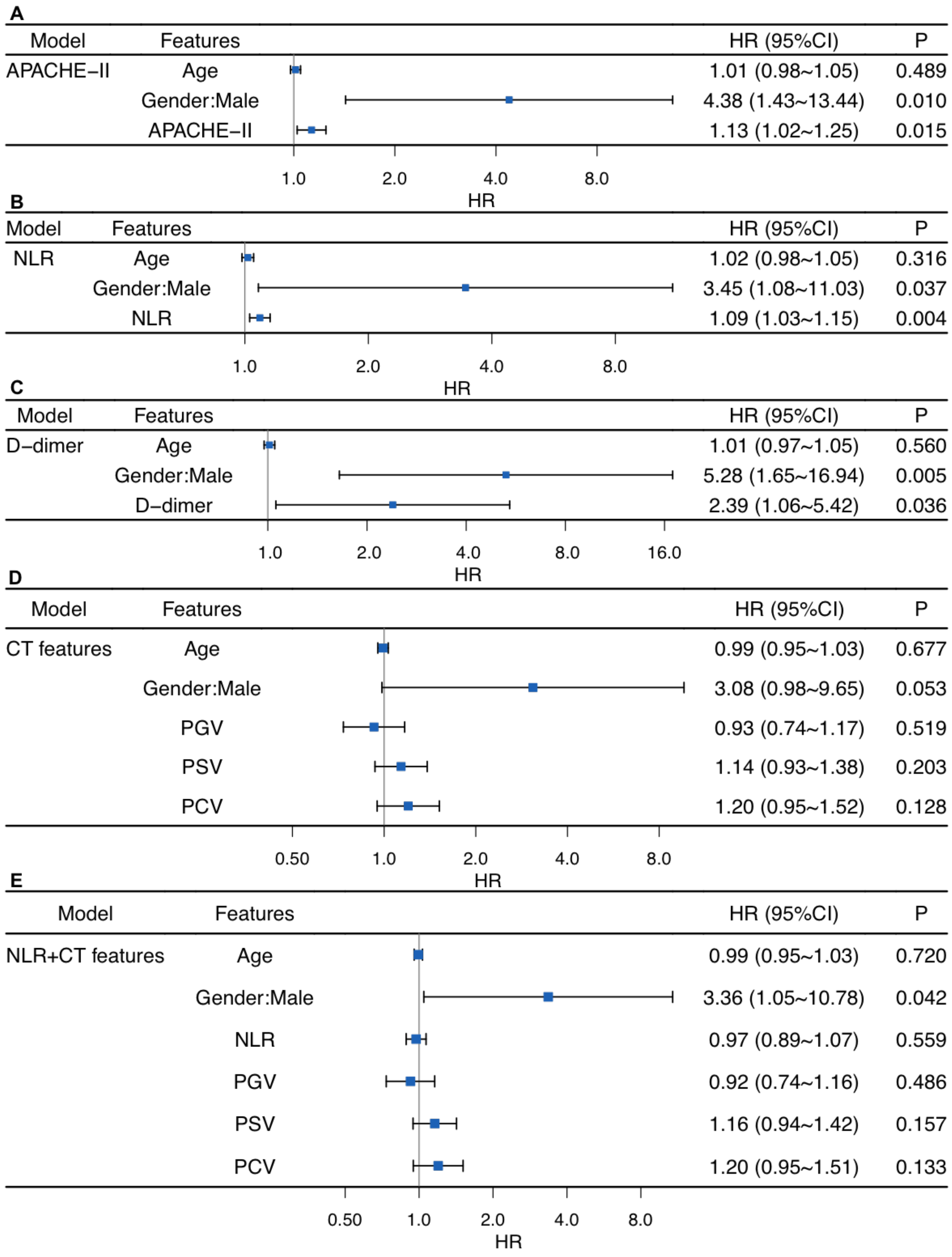

Figure S6. Relationship between each feature on day 4 and severe-event-free survival on the multivariate Cox analyses. All models were adjusted for traditional clinical variables including age and gender.

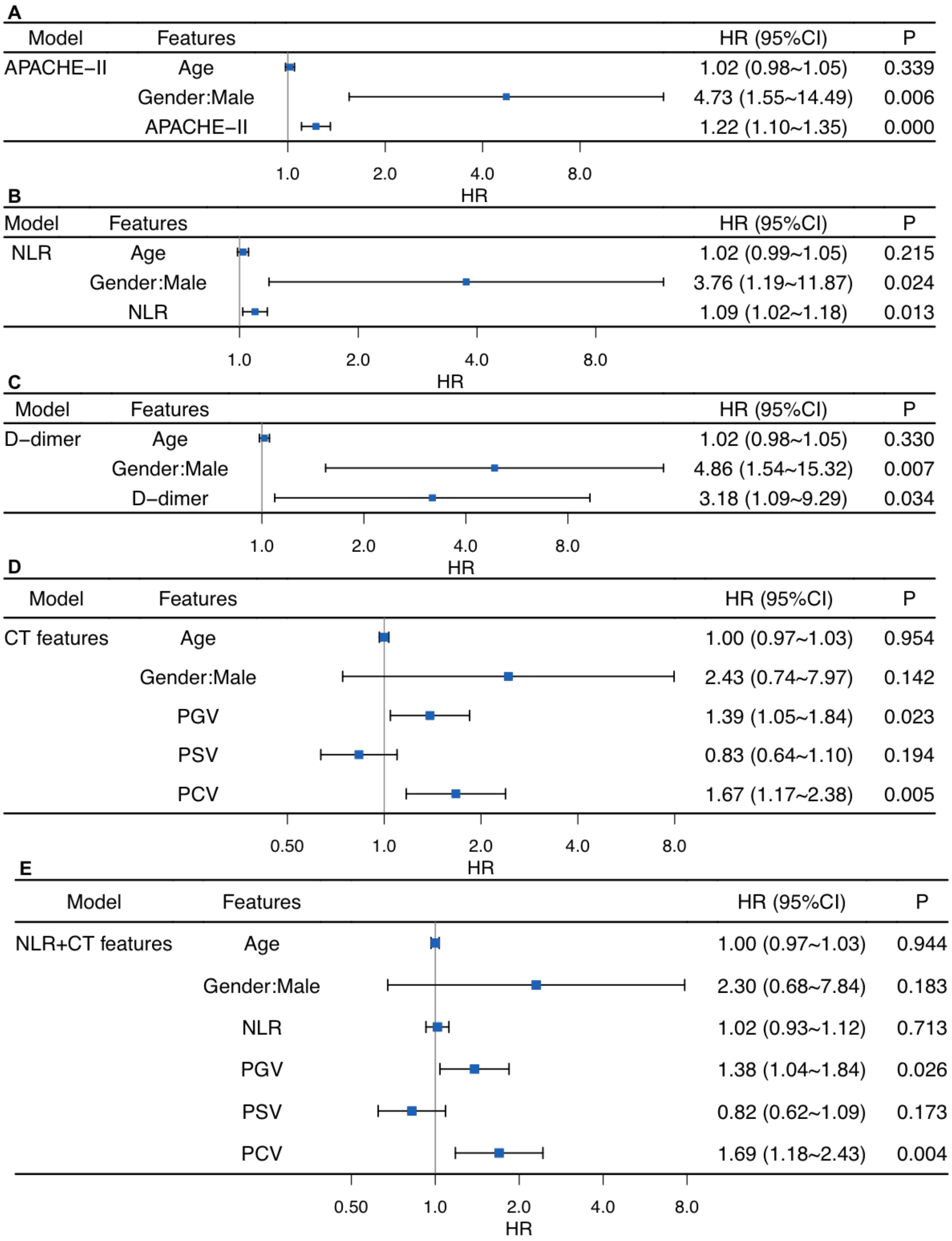

Figure S7. Relationship between changes in each feature from day 0 to day 4 and severe-event-free survival on the multivariate Cox analyses. All models were adjusted for traditional clinical variables including age and gender.
